# Supplementary material for: GuUGT, a glycosyltransferase from Glycyrrhiza uralensis, exhibits glycyrrhetinic acid 3- and 30-O-glycosylation
Source: R Soc Open Sci. 2019 Oct 9;6(10):191121. doi: 10.1098/rsos.191121 (PMC6837211; doi:10.1098/rsos.191121)
Supplement: Supplementary material of this manscuript [file rsos191121supp2.docx]

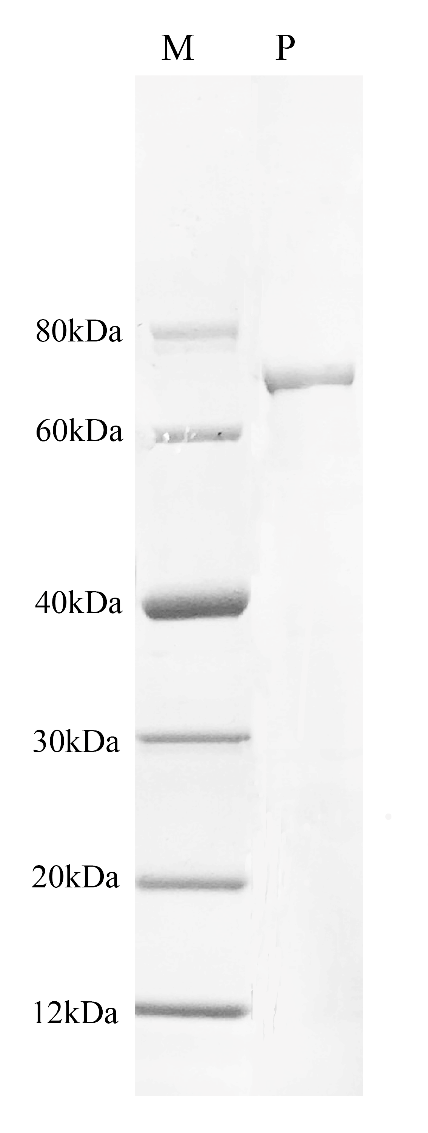
**Figure S1 The chemical structure of UDP-Glc**

**Figure S2. Purification and SDS-PAGE of GuUGT. M, protein marker; P, recombinant GuUGT.**


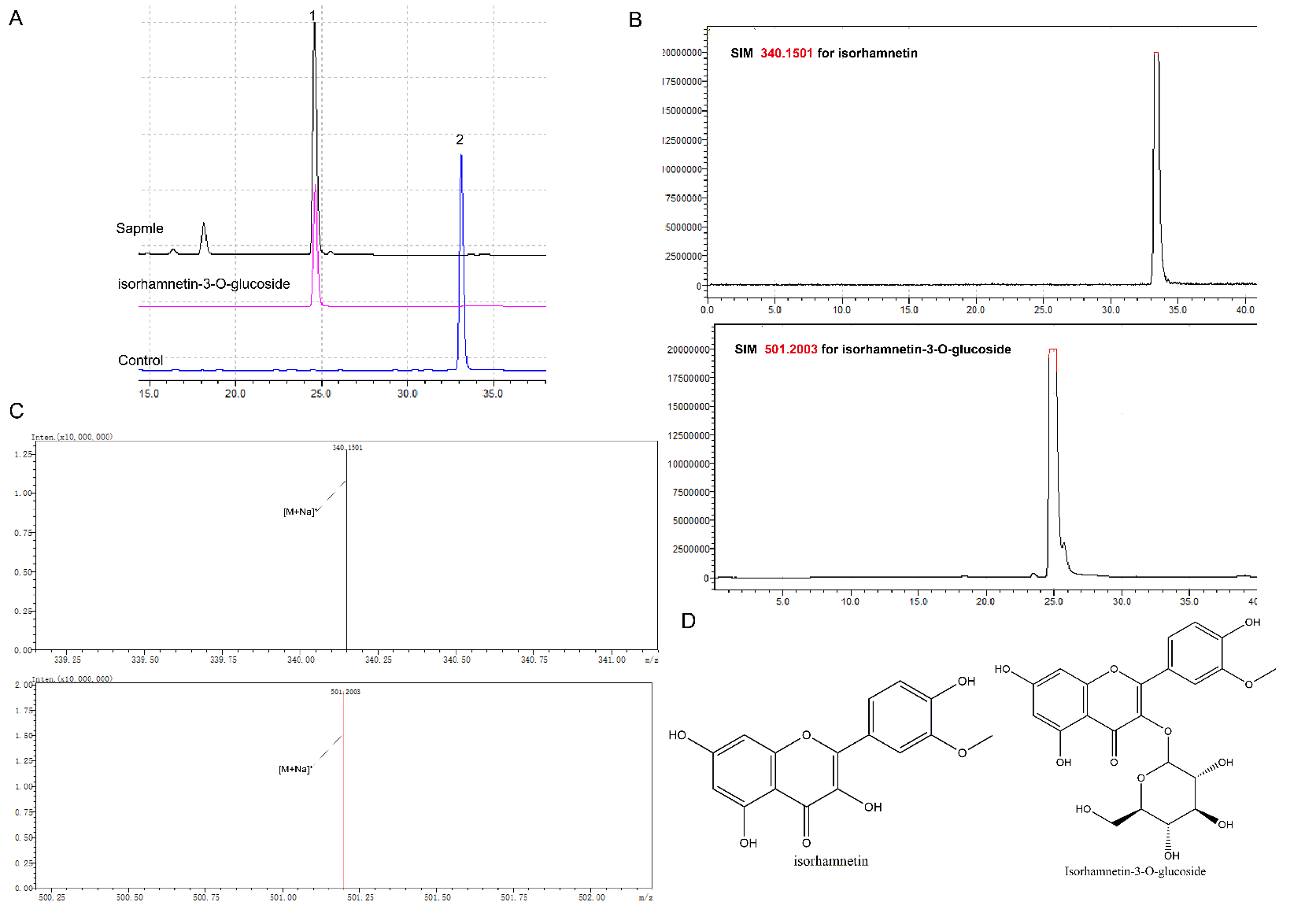


**Figure S3. Glycosylation of** **isorhamnetin the purified glycosyltransferase (recombinant GuUGT). (A) HPLC analysis of the glycosylation products of isorhamnetin catalysed by GuUGT; 1: isorhamnetin-3-O-glucoside; 2: isorhamnetin (B) SIM spectra of the glycosylation products of** **isorhamnetin catalysed by GuUGT (isorhamnetin was m/z 340.1501; isorhamnetin-3-O-glucoside was 501.2003). (C) MS spectra of glycosylation products: isorhamnetin: (m/z 340.1501) isorhamnetin-3-O-glucoside (m/z 501.2003). (D) The chemical structure of isorhamnetin and isorhamnetin-3-O-glucoside.**

**Table S1 Primers for GuUGT cloning**

| **Name** | **Target Gene** | **Sequence (5' To 3')** |
| --- | --- | --- |
| GuUGT-F | GuUGT | ATGGGTAGTAATAGCAATCAAGC |
| GuUGT-R |  | TCAATGGGTAAGTGAACCCAAC |

**Table S2 Primers used for qRT-PCR**

| **Name** | **Sequence (5' To 3')** |
| --- | --- |
| β-actin-F | TGCTCTGGCTCCTAGCAGCATGAAGA |
| β-actin-R | CACATCTGCTGGAAGGTGCTGAGGGA |
| GuUGT-1F | ACTCGAGAAGGCTTATGCGG |
| GuUGT-1R | GCCATTTCAGACACTCGTGC |

**Table S3 Plasmids used in this study**

| **Name** | **description** | **source** |
| --- | --- | --- |
| pδ-VvCPR | Cloning *P_PGK1_-VvCPR-T_ADH1_* cassette into pEASY-Blunt simple | [1] |
| pNDT80-HIS | Cloning *NDT80* Site and *HIS3* marker into pEASY-Blunt | [2] |
| M4-CYP72A63 | Cloning *P_TDH3_-CYP72A63-T_TPI1_* cassette into pEASY-Blunt simple | This study |
| 313-TRP-TEF-CYP88D6 | Cloning *P_TEF1_-GuCYP88d6-T_CYC1_* cassette into 313-TRP | This study |
| M2-AtUDH | Cloning *P_PGK1_-AtUDH-T_ADH1_* cassette into pEASY-Blunt | This study |
| M13-GuUGT | Cloning *P_TEF1_-GuCYP88d6-T_CYC1_* cassette into pEASY-Blunt | This study |

**Table S4 Primers for plasmid construction**

| **Name** | **Target Gene** | **Sequence (5' TO 3')** |
| --- | --- | --- |
| Pac1-88d6 | *Gucyp88d6* | Gcg ttaattaa ATGGAAGTACATTGGGTTTGCATGTCCG |
| 88d6- AscI |  | Gc ggcgcgcc CTAAGCACATGAGACCTTCATCACCTTAGCGA |
| SexA1-72a63 | *opticyp72a63* | Gcg ACCTGGTAAAACA ATGGAAGTTTTTATGTTTCCAACAGGTACT |
| 72a63-asc1 |  | Gc GGCGCGCC TTACAATTTATGCAAAATGATAGATGCACC |
| SexAI-AtUDH | *Atudh* | GCGaccTggtaaaacaATGGTGAAGATATGCTGCATAGGAG |
| AscI-AtUDH |  | GcggcgcgccTCATGCCACAGCAGGCATATCCTTG |
| Pac1-Guugt | *Guugt* | Gcg ttaattaa ATGGGTAGTAATAGCAATCAAGC |
| GuugtC-Asc1 |  | Gc ggcgcgcc TCAATGGGTAAGTGAACCCAAC |
| SexA1-vvcpr | *VvCPR* | GCGaccTggtaaaacaATGCAATCATCCTCCGTAAAGGTAT |
| vvcpr-Asc1 |  | GcggcgcgccTTAGACATCTCTCAAGTATCTACCA |

**Table S5 Primers used for DNA assemble**

| **Name** | **Sequence (5' TO 3')** |
| --- | --- |
| X1-M-pEASY- r-t-F | CTTGCAAATGCCTATTGTGCAGATGTTATAATATCTGTGCGTTTAATTAAGGCTCGTATGTTGTGTGGAATTGT |
| X2-M-pEASY-r-t-R | CGAAGGCTTTAATTTGCAAGCTGCGGCCCTGCATTAATGAATCGGCCAACGCGCCAGGGTTTTCCCAGTCACGACGTTG |
| NDT80-interg-1 | CATCATAAGGAATTCCGGGATTCTCCCCAT |
| NDT80-interg-2 | ACAACATTTGGTCACTAAATCGATATTTTAC |
| 1-M-pEASY-PGK1-F | CTGTTTCCTGTGTGAAATTGTTATCCGCTCACAATTCCACACAACATACGAGCCTTAATTAAACGCACAGATATTATAAC |
| 3G -1-M-ADHt-TDH3-R | CCTCCGCGTCATTAAACTTCTTGTTGTTGACGCTAACATTCAACGCTAGTATTCGGCATGCCGGTAGAGGTGTGG |
| 3G -3-M-ADHt-TDH3-F | CAGGTATAGCATGAGGTCGCTCTTATTGACCACACCTCTACCGGCATGCCGAATACTAGCGTTGAATGTTAGCGTC |
| 3G -3-M-TPI1t-TEF1-R | AGGAGTAGAAACATTTTGAAGCTATGGTGTGTGGGGGATCACTTTAATTAA TCTATATAACAGTTGAAATTTGGA |
| 3G -2-M-TPI1t-TEF1-F | GTCATTTTCGCGTTGAGAAGATGTTCTTATCCAAATTTCAACTGTTATATAGATTAATTAAAGTGATCCCCCACAC |
| M-CYC1t-pEASY-R | CGTATTACAATTCACTGGCCGTCGTTTTACAACGTCGTGACTGGGAAAACCCTGGCGCGTTGGCCGATTCATTAATGC |
| EGH1-UP-F | GGCAACGAGATCCAATTGCGTGGTGTC |
| EGH1-UP-R | CTTAGGGTAGGACTCTTCATCTTTGCC |
| EGH1-Down-F | GCATTAGAAGGAAGTAATCTTTCGTAC |
| EGH1-Down-R | GCAAATTTCAATGAACTGATGGCCAGG |
| EGH1-up-Marker-50bp-F | GACCCCAAAACGGGCAGGGAAGTTGGCAAAGATGAAGAGTCCTACCCTAAGCACACAGGAAACAGCTATGACC |
| Only-pPGK1-Cut-R | CACGAGGTTCTACTAAACTAAACCACC |

**Table S6 All strains in this study**

| **Name** | **description** | **source** |
| --- | --- | --- |
| BY-βA-G | BY-T1, *rDNA::P_PGK1_-GgbAS-T_ADH1_-P_TDH3_-ERG1-T_TPI1_-P_TEF1_-ERG9-T_CYC1_* | [3] |
| BY-UGT | BY-βA-G，*EGH::TRP1- P_PGK1_-AtUDH-T_ADH11_-P_TEF1_-GuUGTC-T_CYC1_*, | This study |
| BY-GA-glucoside derivates | NDT80::HIS3-P*_PGK1_-VvCPR-T_ADH1_-P_TDH3_-A63-T_TPI1_-P_TEF1_-88D-T_CYC1_* | This study |

References

1. Lin, *et al.* Construction of cell factories for production of lupeol in Saccharomyces cerevisiae. *Chinas journal of Chinese Materia medicine* 2018, **41**:1008-1015

2. Dai Z, Liu Y, Sun Z, et al. Identification of a novel cytochrome P450 enzyme that catalyzes the C-2α hydroxylation of pentacyclic triterpenoids and its application in yeast cell factories Metabolic engineering 2019, **51**: 70-78.

3. Dai Z, Wang B, Liu Y, et al. Producing aglycons of ginsenosides in bakers' yeast. Scientific reports, 2014, **4**: 3698.
